# 3-(4-Methoxy­phen­yl)pent-2-ene-1,5-dioic acid

**DOI:** 10.1107/S1600536808037495

**Published:** 2008-11-20

**Authors:** Ushati Das, Shardul B. Chheda, Suhas R. Pednekar, Narendra P Karambelkar, T. N. Guru Row

**Affiliations:** a Solid State and Structural Chemistry Unit, Indian Institute of Science, Bangalore 560 012, India; b F-12, Organic Chemistry Research Laboratory, Ramanujan Ruia College, Matunga (East), Mumbai 400 019, Maharashtra, India; c Jai Research Foundation, C-12, Road No. 16, Wagale Industrial Estate, Thane (West) 400 064, Maharashtra, India

## Abstract

In the title compound, C_12_H_12_O_5_, mol­ecules are linked into anti­parallel hydrogen-bonded sheets through inversion dimers generated *via* two O—H⋯O hydrogen bonds. Using the *R*_2_^2^(8) motif as a building block, hydrogen-bonded chains of a *C*_2_^2^(8) superstructure are then generated.

## Related literature

For 3-(4-methoxy­phen­yl)-2-pentene-1,5-dioic acid as a synthon in organic chemistry, see: Kon & Nanji (1933 ▶); Linstead (1941 ▶). A number of heterocycles such as pyridine-2,6-diones can be obtained from it, see: Pednekar *et al.* (2004 ▶). For hydrogen-bond motifs, see: Bernstein *et al.* (1995 ▶).
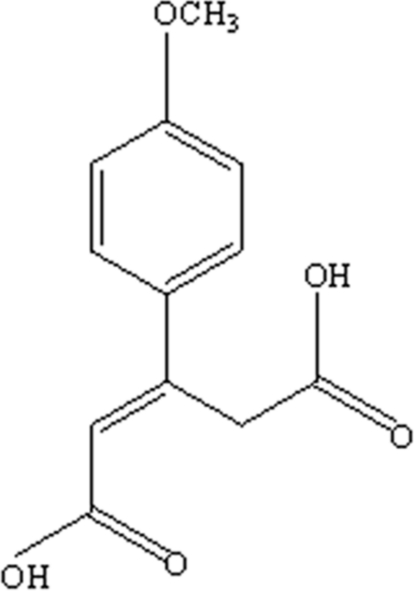


## Experimental

### 

#### Crystal data

C_12_H_12_O_5_
*M* *_r_* = 236.22
Monoclinic, 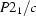

*a* = 5.011 (1) Å
*b* = 10.940 (2) Å
*c* = 20.438 (4) Å
β = 90.665 (3)°
*V* = 1120.3 (4) Å^3^
*Z* = 4
Mo *K*α radiation
μ = 0.11 mm^−1^
*T* = 292 (2) K
0.52 × 0.32 × 0.25 mm

#### Data collection

Bruker SMART APEX CCD area-detector diffractometer
Absorption correction: multi-scan (*SADABS*; Sheldrick, 1996 ▶) *T*_min_ = 0.902, *T*_max_ = 0.973
8509 measured reflections
2190 independent reflections
2140 reflections with *I* > 2σ(*I*)
*R*_int_ = 0.021

#### Refinement

*R*[*F* ^2^ > 2σ(*F* ^2^)] = 0.068
*wR*(*F* ^2^) = 0.150
*S* = 1.22
2190 reflections
157 parameters
H-atom parameters constrained
Δρ_max_ = 0.32 e Å^−3^
Δρ_min_ = −0.29 e Å^−3^

### 

Data collection: *SMART* (Bruker, 2004 ▶); cell refinement: *SAINT* (Bruker, 2004 ▶); data reduction: *SAINT*; program(s) used to solve structure: *SHELXS97* (Sheldrick, 2008 ▶); program(s) used to refine structure: *SHELXL97* (Sheldrick, 2008 ▶); molecular graphics: *ORTEP-3 for Windows* (Farrugia, 1999 ▶) and *CAMERON* (Watkin *et al.*, 1993 ▶); software used to prepare material for publication: *PLATON* (Spek, 2003 ▶).

## Supplementary Material

Crystal structure: contains datablocks global, I. DOI: 10.1107/S1600536808037495/bq2105sup1.cif

Structure factors: contains datablocks I. DOI: 10.1107/S1600536808037495/bq2105Isup2.hkl

Additional supplementary materials:  crystallographic information; 3D view; checkCIF report

## Figures and Tables

**Table 1:** Hydrogen-bond geometry (Å, °)

*D*—H⋯*A*	*D*—H	H⋯*A*	*D*⋯*A*	*D*—H⋯*A*
O1—H1⋯O2^i^	0.82	1.82	2.636 (3)	178
O3—H3⋯O4^ii^	0.82	1.85	2.672 (3)	175
C4—H4*A*⋯O2	0.97	2.19	2.834 (3)	123

Symmetry codes: (i) 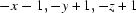; (ii) 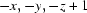.

**Fig. 1.**
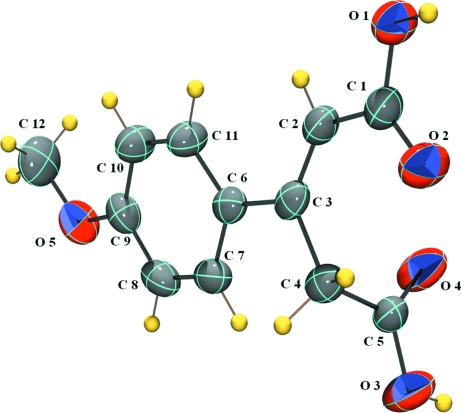
View of the molecular structure of (I) with displacement ellipsoids drawn at the 50% probability level. H atoms are shown as small spheres of arbitrary radius.

**Fig. 2.**
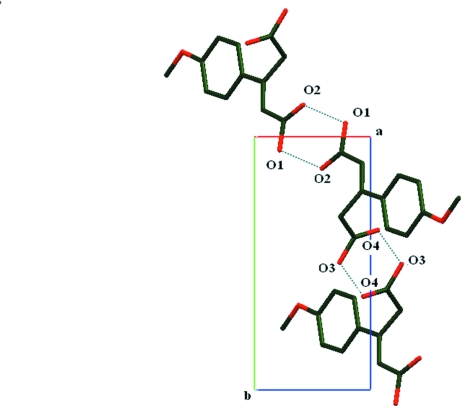
Packing diagram of (I) viewed down the c axis. The dotted lines indicate intermolecular O—H···O interactions.

## supplementary crystallographic information

### Comment

3-(4-Methoxyphenyl)-2-pentene-1,5-dioic acid (Kon & Nanji, 1933; Linstead, 1941) acts as a synthon in organic chemistry. It is a precursor for many heterocyclic ring structures due to its inherent ability to form cyclic anhydrides. A number of heterocycles like pyridine-2,6-diones can be obtained from this dicarboxylic acid (Pednekar *et al.*, 2004). Introduction of double bond at the α,β-position of the glutaric acid, leads to the formation of a glutaconic acid. As a consequence of this, there is a noticeable change in its reactivity. The reactivity of methylene group at γ-position is primarily responsible for the electrophilic substitutions taking places at the γ-position of 3-(4-Methoxyphenyl) -2-pentene-1,5-dioic acid.

The title compound, (I) crystallizes in the monoclinic crystal system in the centrosymmetric space group *P*2_1_/*c* with *Z* = 4. Fig. 1 shows the asymmetric unit and the atom-numbering scheme. Selected bond lengths and torsion angles are given in Table 1.

In the asymmetric unit, compound (I) adopts the *E* conformation, with the =CH-COOH group in the plane of the methoxy phenyl ring (C6—C3—C2—C1 dihedral angle is 174.73°) while the –CH_2_—COOH group lies nearly perpendicular to the phenyl ring (C6—C3—C4—C5 dihedral angle is 89.25 °). Within the aryl rings, the C—C bonds are in the range of 1.374 (3) to 1.395 (3) Å, which is in accordance with those found in similar structures. The C—C single bonds (1.462 (3) to 1.511 (3) Å), C—C double bond (1.339 (3) Å) and C—O double bonds (1.219 (3) Å) are within the usual range.

The molecular assembly is stabilized by extensive intermolecular O—H···O hydrogen bonding, besides intramolecular C—H···O stabilizing weak interactions (Fig. 2). O1 at (*x*,*y*,*z*) acts as a hydrogen-bond donor to O2 at (3 - *x*,2 - *y*,-*z*) to form an inversion dimer centered at (1/2,0,0) and characterized by the usual *R*_2_^2^(8) motif (Bernstein *et al.*, 1995). The inversion dimer acts as a building block and leads to propagation of hydrogen bonded chains to generate a C_2_^2^(8) superstructure. Similarly, O3 (*x*,*y*,*z*) acts as a hydrogen-bond donor to O4 at (2 - *x*,1 - *y*,-*z*) to form an inversion dimer centered at (1,1/2,0), also characterized by the *R*_2_^2^(8) motif. Furthermore, the combination of two inversion dimers nearly perpendicular to one another leads to the formation of an intermolecular hydrogen-bonded staircase with neighboring inversion dimers generated *via* O1—H1···O2 strong hydrogen bonds being connected by O3—H3···O4 interactions. The overall supramolecular packing shows a layered arrangement, with alternate layers mutually parallel.

### Experimental

To synthesize compound (I), citric acid [0.13 mol] was warmed in conc. H_2_SO_4_ (98%) with constant stirring till foam disappeared to obtain acetone dicarboxylic acid. By immersing the reaction flask in an ice bath; temperature was dropped down to 0°C. Anisole [0.113 mol] was then added slowly with vigorous stirring, over a period of one hour. During the addition, temperature was maintained at 0°C. Stirring was continued for a period of 6 hrs, while maintaining the temperature between 0°C to 5°C. The reaction mixture was then poured over crushed ice with stirring. The solid obtained was then filtered and washed with water and colorless single crystals grown from hot water (yield 12%, m.p. 445–447 K). ^1^H NMR (DMSO):δ 12.284 (*s*, 2H), 7.489 (*d*, 2H), 6.951 (*d*, 2H), 6.173 (*s*, 1H), 4.103 (*s*, 2H), 3.768 (*s*, 3H).

### Refinement

All the H atoms were located and refined isotropically resulting in C—H bond lengths of 0.93 (3)–0.97 (3) Å.

### Crystal data

**Table:**

C_12_H_12_O_5_	*F*_000_ = 496
*M**_r_* = 236.22	*D*_x_ = 1.401 Mg m^−^^3^
Monoclinic, *P*2_1_/*c*	Mo *K*α radiation λ = 0.71073 Å
Hall symbol: -P 2ybc	Cell parameters from 2190 reflections
*a* = 5.011 (1) Å	θ = 2.0–26.0º
*b* = 10.940 (2) Å	µ = 0.11 mm^−^^1^
*c* = 20.438 (4) Å	*T* = 292 (2) K
β = 90.665 (3)º	Cylindrical, colourless
*V* = 1120.3 (4) Å^3^	0.52 × 0.32 × 0.25 mm
*Z* = 4	

### Data collection

**Table:**

Bruker SMART APEX CCD area-detector diffractometer	2190 independent reflections
Radiation source: fine-focus sealed tube	2140 reflections with *I* > 2σ(*I*)
Monochromator: graphite	*R*_int_ = 0.021
*T* = 292(2) K	θ_max_ = 26.0º
φ and ω scans	θ_min_ = 2.0º
Absorption correction: multi-scan(SADABS; Sheldrick, 1996)	*h* = −6→6
*T*_min_ = 0.903, *T*_max_ = 0.973	*k* = −13→13
8509 measured reflections	*l* = −23→25

### Refinement

**Table:**

Refinement on *F*^2^	Secondary atom site location: difference Fourier map
Least-squares matrix: full	Hydrogen site location: inferred from neighbouring sites
*R*[*F*^2^ > 2σ(*F*^2^)] = 0.068	H-atom parameters constrained
*wR*(*F*^2^) = 0.151	*w* = 1/[σ^2^(*F*_o_^2^) + (0.0537*P*)^2^ + 0.2183*P*] where *P* = (*F*_o_^2^ + 2*F*_c_^2^)/3
*S* = 1.22	(Δ/σ)_max_ < 0.001
2190 reflections	Δρ_max_ = 0.32 e Å^−^^3^
157 parameters	Δρ_min_ = −0.29 e Å^−^^3^
Primary atom site location: structure-invariant direct methods	Extinction correction: none

### Special details

**Table:**

Geometry. Bond distances, angles *etc*. have been calculated using the rounded fractional coordinates. All su's are estimated from the variances of the (full) variance-covariance matrix. The cell e.s.d.'s are taken into account in the estimation of distances, angles and torsion angles
Refinement. Refinement of *F*^2^ against ALL reflections. The weighted *R*-factor *wR* and goodness of fit *S* are based on *F*^2^, conventional *R*-factors *R* are based on *F*, with *F* set to zero for negative *F*^2^. The threshold expression of *F*^2^ > σ(*F*^2^) is used only for calculating *R*-factors(gt) *etc*. and is not relevant to the choice of reflections for refinement. *R*-factors based on *F*^2^ are statistically about twice as large as those based on *F*, and *R*- factors based on ALL data will be even larger.

### Fractional atomic coordinates and isotropic or equivalent isotropic displacement parameters (Å^2^)

**Table:**

	*x*	*y*	*z*	*U*_iso_*/*U*_eq_	
O1	−0.2159 (4)	0.55290 (16)	0.45189 (9)	0.0564 (6)	
O2	−0.4196 (4)	0.37432 (18)	0.46474 (10)	0.0687 (7)	
O3	−0.2679 (5)	0.0034 (2)	0.44062 (11)	0.0841 (9)	
O4	0.0612 (4)	0.13111 (19)	0.46215 (11)	0.0771 (8)	
O5	0.6191 (4)	0.16520 (18)	0.15123 (9)	0.0627 (7)	
C1	−0.2519 (5)	0.4369 (2)	0.43791 (11)	0.0464 (7)	
C2	−0.0718 (5)	0.3943 (2)	0.38696 (11)	0.0440 (7)	
C3	−0.0755 (4)	0.2846 (2)	0.35786 (11)	0.0405 (7)	
C4	−0.2604 (4)	0.1838 (2)	0.37974 (12)	0.0445 (7)	
C5	−0.1392 (5)	0.1029 (2)	0.43137 (11)	0.0434 (7)	
C6	0.1041 (4)	0.2562 (2)	0.30272 (11)	0.0402 (7)	
C7	0.1417 (5)	0.1368 (2)	0.28065 (12)	0.0490 (8)	
C8	0.3102 (5)	0.1101 (2)	0.23008 (13)	0.0533 (8)	
C9	0.4516 (5)	0.2015 (2)	0.19929 (12)	0.0479 (8)	
C10	0.4146 (5)	0.3214 (2)	0.21898 (13)	0.0517 (8)	
C11	0.2436 (5)	0.3467 (2)	0.26956 (12)	0.0488 (8)	
C12	0.7575 (7)	0.2569 (3)	0.11593 (16)	0.0731 (11)	
H1	−0.33195	0.57564	0.47710	0.0845*	
H2	0.05853	0.44896	0.37338	0.0528*	
H3	−0.19480	−0.03596	0.46989	0.1262*	
H4A	−0.42219	0.22012	0.39662	0.0534*	
H4B	−0.30956	0.13424	0.34214	0.0534*	
H7	0.04987	0.07345	0.30076	0.0587*	
H8	0.32931	0.02950	0.21641	0.0639*	
H10	0.50463	0.38443	0.19819	0.0621*	
H11	0.22033	0.42766	0.28206	0.0586*	
H12A	0.63099	0.31041	0.09496	0.1099*	
H12B	0.86702	0.21914	0.08343	0.1099*	
H12C	0.86800	0.30320	0.14555	0.1099*	

### Atomic displacement parameters (Å^2^)

**Table:**

	*U*^11^	*U*^22^	*U*^33^	*U*^12^	*U*^13^	*U*^23^
O1	0.0599 (11)	0.0484 (10)	0.0612 (11)	−0.0047 (8)	0.0206 (9)	0.0003 (8)
O2	0.0753 (13)	0.0570 (11)	0.0744 (13)	−0.0160 (10)	0.0334 (11)	−0.0041 (9)
O3	0.0967 (16)	0.0679 (13)	0.0869 (16)	−0.0389 (12)	−0.0369 (13)	0.0386 (11)
O4	0.0760 (14)	0.0718 (13)	0.0828 (14)	−0.0282 (11)	−0.0339 (12)	0.0371 (11)
O5	0.0659 (12)	0.0635 (12)	0.0590 (11)	0.0089 (9)	0.0095 (9)	−0.0162 (9)
C1	0.0473 (13)	0.0478 (13)	0.0440 (12)	−0.0040 (10)	0.0020 (10)	0.0071 (10)
C2	0.0465 (13)	0.0430 (12)	0.0427 (12)	−0.0048 (10)	0.0041 (10)	0.0076 (10)
C3	0.0376 (11)	0.0424 (12)	0.0412 (12)	−0.0005 (9)	−0.0060 (9)	0.0119 (9)
C4	0.0398 (12)	0.0434 (12)	0.0503 (13)	−0.0038 (10)	−0.0024 (10)	0.0054 (10)
C5	0.0468 (13)	0.0424 (12)	0.0410 (12)	−0.0089 (10)	0.0003 (10)	0.0056 (9)
C6	0.0401 (11)	0.0387 (11)	0.0417 (12)	0.0023 (9)	−0.0058 (9)	0.0043 (9)
C7	0.0551 (14)	0.0399 (12)	0.0517 (14)	−0.0056 (10)	−0.0068 (11)	0.0004 (10)
C8	0.0620 (15)	0.0406 (13)	0.0569 (15)	0.0041 (11)	−0.0096 (12)	−0.0104 (11)
C9	0.0455 (13)	0.0532 (14)	0.0448 (13)	0.0077 (11)	−0.0041 (10)	−0.0082 (11)
C10	0.0545 (14)	0.0431 (13)	0.0578 (15)	0.0008 (11)	0.0128 (12)	0.0006 (11)
C11	0.0542 (14)	0.0350 (11)	0.0575 (14)	0.0025 (10)	0.0104 (11)	0.0005 (10)
C12	0.0732 (19)	0.082 (2)	0.0647 (18)	0.0137 (16)	0.0231 (15)	−0.0056 (16)

### Geometric parameters (Å, °)

**Table:**

O1—C1	1.313 (3)	C7—C8	1.374 (4)
O2—C1	1.219 (3)	C8—C9	1.381 (3)
O3—C5	1.280 (3)	C9—C10	1.385 (3)
O4—C5	1.219 (3)	C10—C11	1.378 (4)
O5—C9	1.359 (3)	C2—H2	0.9300
O5—C12	1.421 (4)	C4—H4A	0.9700
O1—H1	0.8200	C4—H4B	0.9700
O3—H3	0.8200	C7—H7	0.9300
C1—C2	1.462 (3)	C8—H8	0.9300
C2—C3	1.339 (3)	C10—H10	0.9300
C3—C6	1.483 (3)	C11—H11	0.9300
C3—C4	1.511 (3)	C12—H12A	0.9600
C4—C5	1.500 (3)	C12—H12B	0.9600
C6—C11	1.393 (3)	C12—H12C	0.9600
C6—C7	1.395 (3)		
			
O1···O5^i^	3.152 (3)	C10···H12A	2.7700
O1···C1^ii^	3.234 (3)	C10···H12C	2.7400
O1···O1^ii^	3.129 (3)	C10···H8^vi^	2.9200
O1···O2^iii^	2.636 (3)	C11···H2	2.5800
O2···O1^iii^	2.636 (3)	C11···H8^vi^	2.9400
O2···C4	2.834 (3)	C12···H10	2.5400
O2···C5	3.358 (3)	H1···O2^iii^	1.8200
O2···C1^iii^	3.318 (3)	H1···C1^iii^	2.7300
O2···O2^iii^	3.212 (3)	H1···H1^iii^	2.5500
O3···O4^iv^	2.672 (3)	H2···C11	2.5800
O4···C5^iv^	3.380 (3)	H2···H11	2.0600
O4···O3^iv^	2.672 (3)	H2···O5^vi^	2.9100
O4···C2	3.328 (3)	H3···O4^iv^	1.8500
O5···O1^v^	3.152 (3)	H3···C5^iv^	2.7100
O1···H12B^vi^	2.6300	H3···H3^iv^	2.4300
O2···H4A	2.1900	H4A···O2	2.1900
O2···H1^iii^	1.8200	H4A···C1	2.6500
O3···H12A^v^	2.8800	H4A···C6^ix^	3.0600
O4···H3^iv^	1.8500	H4B···C7^ix^	3.0100
O5···H2^vii^	2.9100	H4B···C7	2.6000
C1···O1^ii^	3.234 (3)	H4B···C8^ix^	2.9700
C1···O2^iii^	3.318 (3)	H4B···H7	2.1100
C2···C8^i^	3.558 (3)	H7···C4	2.5600
C2···O4	3.328 (3)	H7···C5	2.8600
C4···O2	2.834 (3)	H7···H4B	2.1100
C5···O2	3.358 (3)	H8···C2^v^	2.8700
C5···O4^iv^	3.380 (3)	H8···C10^vii^	2.9200
C5···C7	3.422 (3)	H8···C11^vii^	2.9400
C7···C5	3.422 (3)	H8···H10^vii^	2.4900
C8···C10^vii^	3.596 (3)	H8···H11^vii^	2.5200
C8···C2^v^	3.558 (3)	H10···C12	2.5400
C10···C8^vi^	3.596 (3)	H10···H12A	2.3500
C1···H4A	2.6500	H10···H12C	2.3000
C1···H1^iii^	2.7300	H10···C8^vi^	3.0100
C2···H8^i^	2.8700	H10···H8^vi^	2.4900
C2···H11	2.6400	H11···C2	2.6400
C4···H7	2.5600	H11···H2	2.0600
C5···H3^iv^	2.7100	H11···C8^vi^	3.1000
C5···H7	2.8600	H11···H8^vi^	2.5200
C6···H4A^viii^	3.0600	H12A···C10	2.7700
C7···H4B	2.6000	H12A···H10	2.3500
C7···H4B^viii^	3.0100	H12A···O3^i^	2.8800
C8···H10^vii^	3.0100	H12B···O1^vii^	2.6300
C8···H11^vii^	3.1000	H12C···C10	2.7400
C8···H4B^viii^	2.9700	H12C···H10	2.3000
			
C9—O5—C12	118.0 (2)	C6—C11—C10	122.8 (2)
C1—O1—H1	109.00	C1—C2—H2	117.00
C5—O3—H3	109.00	C3—C2—H2	117.00
O1—C1—C2	112.3 (2)	C3—C4—H4A	109.00
O2—C1—C2	125.2 (2)	C3—C4—H4B	109.00
O1—C1—O2	122.6 (2)	C5—C4—H4A	109.00
C1—C2—C3	126.6 (2)	C5—C4—H4B	109.00
C2—C3—C6	121.3 (2)	H4A—C4—H4B	108.00
C2—C3—C4	121.9 (2)	C6—C7—H7	119.00
C4—C3—C6	116.84 (19)	C8—C7—H7	119.00
C3—C4—C5	113.20 (18)	C7—C8—H8	120.00
O3—C5—C4	113.9 (2)	C9—C8—H8	120.00
O4—C5—C4	122.6 (2)	C9—C10—H10	120.00
O3—C5—O4	123.6 (2)	C11—C10—H10	120.00
C7—C6—C11	116.0 (2)	C6—C11—H11	119.00
C3—C6—C7	121.8 (2)	C10—C11—H11	119.00
C3—C6—C11	122.2 (2)	O5—C12—H12A	109.00
C6—C7—C8	121.9 (2)	O5—C12—H12B	109.00
C7—C8—C9	120.8 (2)	O5—C12—H12C	109.00
O5—C9—C10	124.9 (2)	H12A—C12—H12B	109.00
O5—C9—C8	116.2 (2)	H12A—C12—H12C	109.00
C8—C9—C10	118.8 (2)	H12B—C12—H12C	109.00
C9—C10—C11	119.6 (2)		
			
C12—O5—C9—C8	176.7 (2)	C3—C4—C5—O3	−166.2 (2)
C12—O5—C9—C10	−3.4 (4)	C3—C4—C5—O4	14.9 (3)
O1—C1—C2—C3	−173.4 (2)	C3—C6—C7—C8	−179.3 (2)
O2—C1—C2—C3	5.9 (4)	C11—C6—C7—C8	1.2 (3)
C1—C2—C3—C4	−5.7 (4)	C3—C6—C11—C10	178.9 (2)
C1—C2—C3—C6	174.8 (2)	C7—C6—C11—C10	−1.6 (4)
C2—C3—C4—C5	−90.4 (3)	C6—C7—C8—C9	0.5 (4)
C6—C3—C4—C5	89.2 (2)	C7—C8—C9—O5	178.0 (2)
C2—C3—C6—C7	166.9 (2)	C7—C8—C9—C10	−1.9 (4)
C2—C3—C6—C11	−13.6 (3)	O5—C9—C10—C11	−178.4 (2)
C4—C3—C6—C7	−12.7 (3)	C8—C9—C10—C11	1.5 (4)
C4—C3—C6—C11	166.8 (2)	C9—C10—C11—C6	0.3 (4)

Symmetry codes: (i) −*x*, *y*+1/2, −*z*+1/2; (ii) −*x*, −*y*+1, −*z*+1; (iii) −*x*−1, −*y*+1, −*z*+1; (iv) −*x*, −*y*, −*z*+1; (v) −*x*, *y*−1/2, −*z*+1/2; (vi) −*x*+1, *y*+1/2, −*z*+1/2; (vii) −*x*+1, *y*−1/2, −*z*+1/2; (viii) *x*+1, *y*, *z*; (ix) *x*−1, *y*, *z*.

### Hydrogen-bond geometry (Å, °)

**Table:**

*D*—H···*A*	*D*—H	H···*A*	*D*···*A*	*D*—H···*A*
O1—H1···O2^iii^	0.82	1.82	2.636 (3)	178
O3—H3···O4^iv^	0.82	1.85	2.672 (3)	175
C4—H4A···O2	0.97	2.19	2.834 (3)	123

Symmetry codes: (iii) −*x*−1, −*y*+1, −*z*+1; (iv) −*x*, −*y*, −*z*+1.
